# Detection and characterization of tigecycline heteroresistance in *E. cloacae*: clinical and microbiological findings

**DOI:** 10.1080/22221751.2019.1601031

**Published:** 2019-04-04

**Authors:** Hang Liu, Xiaojiong Jia, Hua Zou, Shan Sun, Shuang Li, Yonghong Wang, Yun Xia

**Affiliations:** Department of Clinical Laboratory, the First Affiliated Hospital of Chongqing Medical University, Chongqing, People’s Republic of China

**Keywords:** Tigecycline heteroresistance, carbapenem-resistant, *Enterobacter cloacae*, efflux pump, risk factors

## Abstract

Tigecycline is regarded as a last-resort treatment for carbapenem-resistant *Enterobacteriaceae* (CRE), however, the emergence of tigecycline heteroresistance has posted the therapeutic challenge to combat this “nightmare bacteria”. The primary purpose of this study was to demonstrate the existence of tigecycline heteroresistance in carbapenem-resistant *E. cloacae* (TH-CRECL) and further to explore the epidemiological characteristics and underlying molecular mechanisms. Our study identified a relative low prevalence of carbapenem-resistant *E. cloacae* (CRECL) isolates, about 20.0% (28/140), as heteroresistance to tigecycline. Molecular genetic relatedness of these heteroresistant isolates were characterized epidemiologically sporadic. In addition, mechanistic analysis revealed that Phe-Arg-β-naphthylamide (PAβN) significantly reversed tigecycline MIC levels of resistant colonies in heteroresistant strains, as primarily related to the marked overproduction of efflux pump genes *acrAB* and *oqxAB*, as well as overexpression of transcriptional regulators (*soxS* and *ramA*). Moreover, logistic regression analysis showed that previous fluoroquinolone therapy was identified as the only potential independent risk factor for the acquisition of TH-CRECL. Most importantly, our data indicated that patients with TH-CRECL infection might lead to a remarkably prolonged hospital stay and deterioration in functional status. These findings emphasized the necessity of timely detection and intervention of patients infected with TH-CRECL.

## Introduction

*Enterobacter cloacae* (*E. cloacae*) has been acknowledged as an important and opportunistic pathogen responsible for a broad range of hospital-acquired infections [1]. The rapid increase in these infections caused by carbapenem-resistant *E. cloacae* (CRECL) has raised growing concern worldwide, which definitely necessitates the availability of ideal antimicrobial agents [2–4]. Tigecycline and colistin exhibit satisfactory activities *in vitro* against serious infections caused by these pathogens [5]. Current evidence suggests that colistin has a narrow therapeutic window, and parenteral administration of colistin has the potential adverse effects of neurotoxicity and nephrotoxicity [6], thus making that tigecycline could be considered as a last-resort drug to treat these serious CRECL infections, especially in some countries where colistin is not commercially available, including in China.

Tigecycline, the first member of glycylcycline antibacterial agents, displays excellent activity against a variety of clinically “difficult-to-treat” microorganisms [7]. Similar to some older tetracyclines, tigecycline can reversibly bind to bacterial 30S ribosomes to halt protein biosynthesis, ultimately limiting the bacterial growth. The main mechanism of tigecycline resistance is the upregulated expression of resistance-nodulation-division (RND) efflux systems, such as, AcrAB in *Escherichia coli* [8], AcrAB together with OqxAB in *Klebsiella pneumonia* and *E. cloacae* [9], as well as AdeABC in *Acinetobacter baumanni* [10]. In addition, overexpressed global transcriptional regulators of these efflux pumps, such as *ramA*, *marA*, and *soxS*, also occupied an irreplaceable position in tigecycline resistance [11,12]. However, tigecycline non-susceptibility in clinical strains could be underestimated when performing the traditional susceptibility testing *in vitro*, possibly due to heteroresistance, which can be defined as the appearance of a resistant division within entirely susceptible germs. Clinical significance of this phenomenon could be considerable, because less susceptible subpopulations might be picked under antibiotic treatment. More importantly, tigecycline is considered as a promising therapeutic option for infections caused by CRE [13]. Emerging tigecycline heteroresistance in CRE posts a diagnostic and therapeutic dilemma for the clinicians, thus further reducing the availability of ideal antibiotic agents to combat this “nightmare bacteria”. To date, information regarding the tigecycline heteroresistance in CRE remains elusive. Our previous study revealed that, among all the CRE isolates collected in our region, the highest carbapenem resistance rate was observed in *E. cloacae* [3]. Therefore, this present study was initiated to explore into the tigecycline heteroresistance in this species. Moreover, no studies have focused on the mechanisms of tigecycline heteroresistance among clinical carbapenem-resistant *E. cloacae* isolates. In addition, data on the identification of patients with TH-CRECL infection may potentially help the clinicians in deciding upon antibiotic therapies and effectively reduce the burden of tigecycline heteroresistance in clinical settings.

Accordingly, the purposes of our investigation were as follows: (i) to confirm the prevalence of tigecycline heteroresistance among the carbapenem-resistant *E. cloacae* strains isolated from nosocomial infections; (ii) to investigate the molecular epidemiology of these TH-CRECL strains; (iii) to explore the underlying mechanisms of parental strains and their resistant subpopulations in the TH-CRECL infection; (iv) to identify the potential risk factors and clinical outcomes of patients with TH-CRECL infection.

## Materials and methods

### Study setting and strains collection

A retrospective analysis of tigecycline heteroresistance in carbapenem-resistant *E. cloacae* was conducted between January 2014 and December 2017. We had collected 140 non-repetitive and tigecycline-susceptible carbapenem-resistant *E. cloacae* isolated from patients with various clinical infections in the first affiliated hospital of Chongqing Medical University and two branch hospitals in Southwest China. The VITEK2 compact or VITEK MS automated system (bioMerieux, Hazelwood, MO, United States) was applied for identification of bacterial species and measurement of minimum inhibitory concentration (MIC) values for different antibiotics. According to the recommended guidelines of the Clinical and Laboratory Standards Institute (CLSI, M100-S27), the tigecycline MIC levels of CRECL isolates were further determined by standard broth microdilution methods. The breakpoint criteria of MICs for tigecycline was based on the latest FDA-Recognized Susceptibility Test Interpretive Criteria, ≤2.0 mg/L as susceptible, 4.0 mg/L as intermediate, and ≥8.0 mg/L as resistant. The *E. cloacae* ATCC 13047 was used as a reference strain. Disk diffusion containing 15μg of tigecycline was placed on Mueller-Hinton agar (MHA) coated with 0.5 McFarland standard bacteria suspension for detection of tigecycline heteroresistance. After 24 h of incubation at 35°C, the obviously visible growth of subpopulations within the inhibitory zone was defined as tigecycline heteroresistance in carbapenem-resistant *E. cloacae* (TH-CRECL), while the absence of colonies within inhibition halo were considered as non-tigecycline heteroresistance in carbapenem-resistant *E. cloacae* (NTH-CRECL).

### PAP analysis and passage stability

PAP (population analysis profile), a gold standard method for determining heteroresistance, was conducted according to our previously reported protocol [14]. Briefly, an aliquot of 100 microliters of 10-fold serially diluted 0.5 McFarland suspension was plated onto MHA plates with tigecycline concentrations of two-fold change gradient from 0.125 to 64 mg/L. Following 48 h of incubating at 37°C, the amounts of colonies were calculated. The analysis was conducted in three replicates. To assess the passage stability of the heteroresistant phenotype, single independent subpopulation growing closest to the paper disk were randomly selected to subculture repeatedly in antibiotic-free medium for over two weeks, and the tigecycline MICs and disk diffusion test were re-performed.

### Growth curve and time–killing assay

Growth curve assays were performed as previously described [15]. Basically, bacteria grew overnight to reach plateau. Then, a 1:100 dilution of saturated culture was mixed into fresh Luria–Bertani broth and shaken at 37°C at 150 rpm. The absorbance of the bacterial medium at 600 nm was determined each hour. The tests were repeated three times, and the means absorbance were applied to calculate the exponential growth rates of the native heteroresistant strains and its resistant subpopulations. The time-killing assays were performed on randomly selected five TH-CRECL strains, on the basis of previously published paper [16].

### Detection of antibiotic-resistant genes and efflux pump inhibitory assay

All the TH-CRECL strains were detected the common carbapenemase-related genes including *NDM*, *KPC*, *VIM*, *SME*, *OXA-48,and IMP*, and ESBLs genes including *TEM*, *SHV*, *CTXM* and *OXA-1* genes by using primers as reported previously [17]. To assess the role of efflux pumps in TH-CRECL parental strains and their resistant subpopulations, the recognized efflux pump inhibitors (EPI) carbonyl cyanide m-chlorophenylhydrazone (CCCP, 16 mg/L, Sigma) [9] and Phe-Arg-β-naphthylamide (PAβN, 20 mg/L, Sigma) [18] were used to inhibit efflux function at tigecycline MIC values by the standard broth microdilution method. Compared with the absence of EPI, the MIC value of tigecycline was decreased by at least 4 times after the addition of EPI, which was considered to be a significant suppression of the efflux pumps [9].

### Quantification of efflux pumps genes

In the native strains and their respective resistant subpopulations, qRT-PCR assays were performed to determine the expression of RND efflux systems related some efflux genes including *acrA*, *acrB*, *oqxA*, and *oqxB*, and their transcriptional regulatory genes *ramA* and *soxS*. All tested strains had positive results in EPI assays as mentioned above, and primer sequences were displayed in Table S1. The relative expression level of each target gene was analyzed according to the 2^−ΔΔCT^ method [19]. The housekeeping gene *rpoB* was used as an internal reference gene, and the expression level of each target gene in the resistant subpopulations were compared to the corresponding gene expression of their respective native strain.

### Risk factors and clinical outcomes of TH-CRECL infection

We analyzed the host-related factors and clinical outcomes of patients with TH-CRECL infection through a retrospective case-control study. All hospitalized patients with positive cultures of TH-CRECL were considered as case group, while patients infected with NTH-CRECL were defined as the control group. We collected the detailed characteristics of infected patients from medical records and microbiological databases, including age, gender, transfer from other hospital, 30-day readmission, admission to ICU, primary diseases and conditions, medical invasions within one month, previous antibiotic therapies within three months, as well as some parameters related to the patients’ clinical outcomes.

### Molecular epidemiological study

PFGE was performed as previously described in all the TH-CRECL strains, and banding patterns were interpreted according to the recommended criteria [16,20]. Similarly, multi-site sequence typing (MLST) was performed by sequencing of seven reference genes and analyzed for all TH-CRECL isolates on the *E. cloacae* MLST database (https://pubmlst.org/ecloacae/).

### Statistical analysis

To determine the potential factors for acquiring the TH-CRECL traits, a univariate analysis was conducted by comparing the TH-CRECL and NTH-CRECL groups. The comparisons of categorical variables were presented as frequency and percentage by using Fisher’s exact test or Chi-square test. The calculations of continuous variables were expressed as a median and interquartile range (IQR) using two simple *t*-test or Wilcoxon rank sum test, depending on whether the data is normal distribution. All variables with a *P* value <0.10 in a univariate analysis were incorporated into a risk factor model. Additionally, the final selected model was tested for confounding and collinearity. If a covariate affected the β-coefficient of a variable in the model by >10%, then the confounding variable was maintained in the multivariate model. The odds ratio (OR) and their 95% confidence interval (CI) were calculated to assess the reliability of each potential predictor. For all analyses, a *P* value <0.05 was defined to be statistically significant. All statistical calculations were conducted with SPSS v.21.0 software (SPSS Inc., Chicago, IL, USA).

### Ethical considerations

This retrospective cohort study was reviewed and approved by the Biomedical Ethics Committee of the First Affiliated Hospital of Chongqing Medical University.

## Results

### First identification of TH-CRECL strains

**Figure 1.**
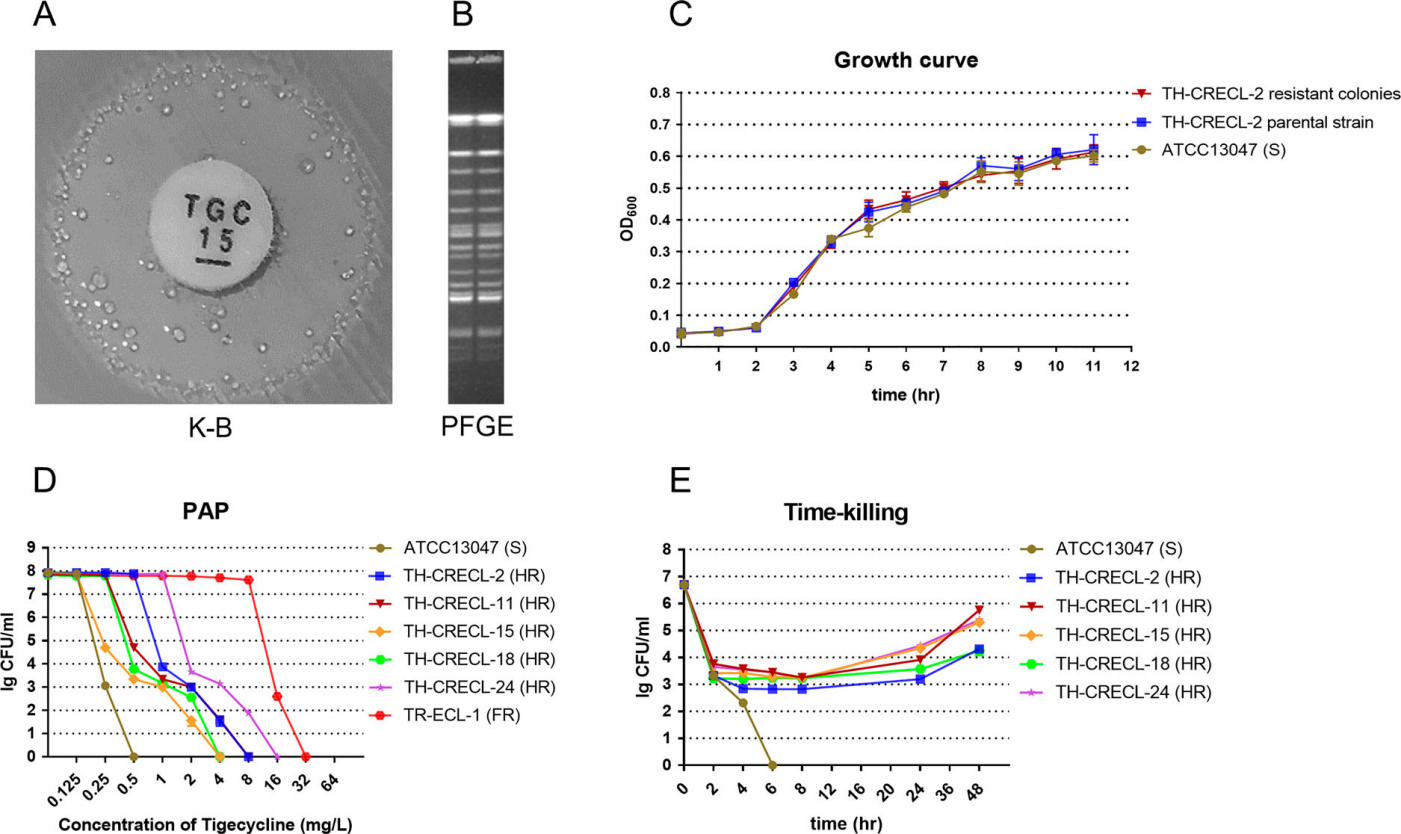
Verification of tigecycline heteroresistance in carbapenem-resistant *E. cloacae* strains. (A) The tigecycline-heteroresistant phenomenon of carbapenem-resistant *E. cloacae* isolates detected by the disk diffusion method. (B) Pulsed-field gel electrophoresis (PFGE) results of the parental strains and subpopulations within the inhibition zones around the tigecycline disks. (C) Growth curve assays for heteroresistant parental isolates and their resistant colonies selected from highest tigecycline concentration agar plates, in the presence or absence of 2 mg/L tigecycline. (D) Population analysis profiles (PAP) of the tigecycline heteroresistant isolates. (E) Time–killing assays for tigecycline heteroresistant isolates. The control strain is *E. cloacae* ATCC 13047. FR, fully tigecycline-resistant and carbapenem-resistant *E. cloacae* (Tigecycline MIC = 32mg/L).

**Figure 2.**
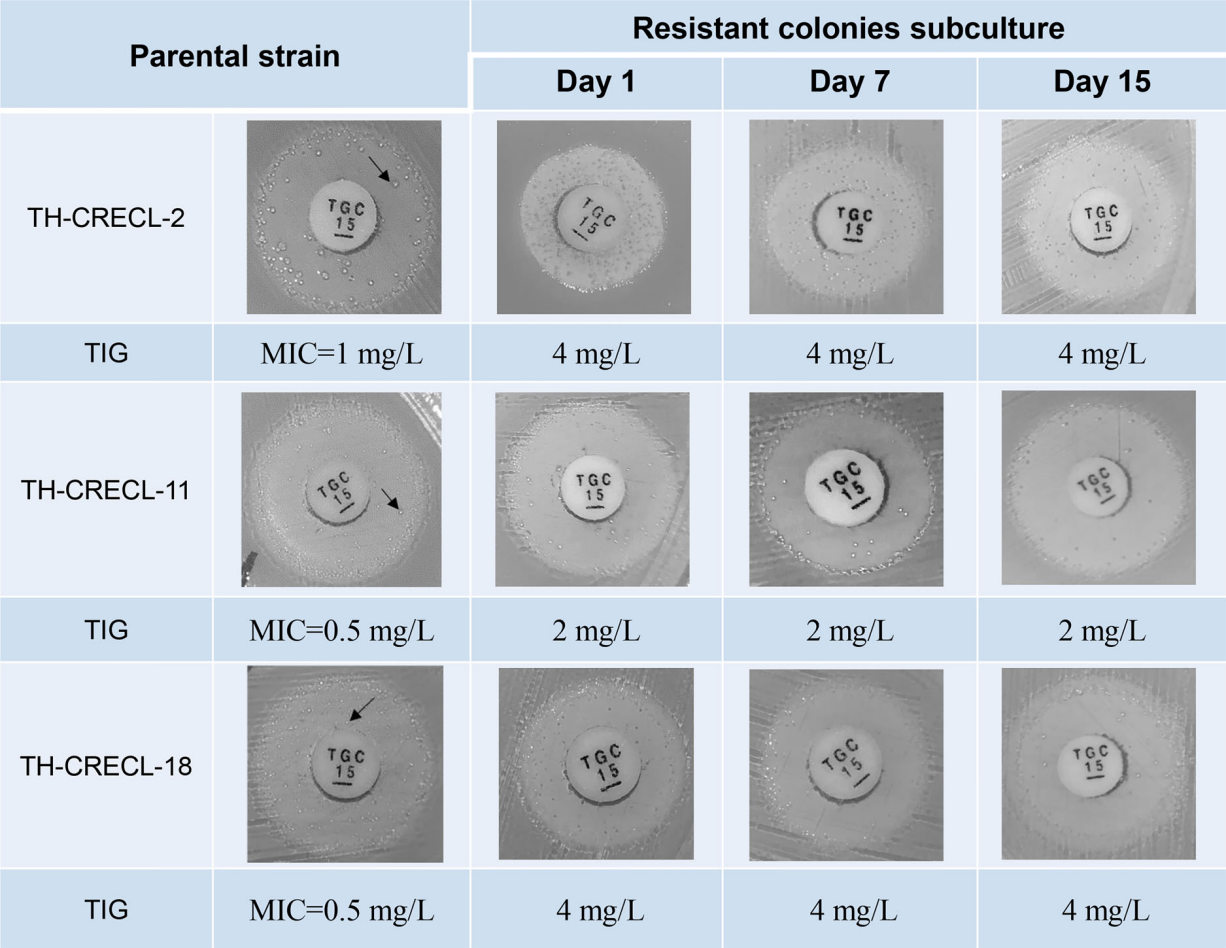
Stability of tigecycline heteroresistance in carbapenem-resistant *E. cloacae* strains. Black arrows indicate selected resistant colonies of subculture.

In this work, we first found the presence of tiny and scattered colonies in the clear zone of inhibition surrounding the tigecycline disks in CRECL isolates (Figure 1A). Additionally, the original strains and their resistant subpopulations displayed similar PFGE band patterns (Figure 1B), indicating that they were isogenic. In addition, the growth kinetics of native cells and their tigecycline-resistant homologous populations showed a rapid growth in the absence of any selective pressure, suggesting that subpopulations of heteroresistant isolates were distinct from the bacteria tolerance that exhibited a slow growth trait [21]. PAP assays confirmed the resistant subcolonies of all five randomly selected heteroresistant strains could grow in tigecycline concentrations at high as 16 mg/L (Figure 1D). In bactericidal kinetic assay, we observed a temperate killing effect on 4 × MIC that was followed by an appreciable re-growth after longer incubation (≥24 h), but the control isolate (*E. cloacae* ATCC 13047) was quickly eliminated (Figure 1E). It suggested that a small number of heteroresistant colonies could not be inhibited under the pressure of tigecycline. Notably, the resistant subcolonies exhibited a minimum of the four-times ascension of tigecycline MIC values compared to their original strains (Table 1). Furthermore, after subculture for 15 days in antibiotic-free medium, the selected strains maintained the stable stage of tigecycline resistance and heteroresistant phenomenon (Figure 2). Collectively, our results revealed the existence of tigecycline heteroresistance in clinical carbapenem-resistant *E. cloacae*.

**Table 1.** Tigecycline MICs in the absence or presence of efflux pump inhibitors in 28 tigecycline-heteroresistant and carbapenem-resistant clinical *E. cloacae* and its resistant subpopulations.

Isolates	Parental strain				Resistant subpopulations^d^			
	TIG^a^	+CCCP^b^	+PAβN^b^	Fold-change^c^	TIG^a^	+CCCP^b^	+PAβN^b^	Fold-change^c^
TH-CRECL-1	1	1	0.25	**4**	8	8	0.25	**32**
TH-CRECL-2	1	1	0.125	**8**	8	8	0.5	**16**
TH-CRECL-3	2	2	1	2	8	8	4	2
TH-CRECL-4	0.25	0.25	0.125	2	2	2	1	2
TH-CRECL-5	1	1	0.25	**4**	8	8	0.25	**32**
TH-CRECL-6	0.5	0.5	0.25	2	4	4	2	2
TH-CRECL-7	0.5	0.5	0.125	4	2	2	1	2
TH-CRECL-8	1	1	0.5	2	4	4	4	1
TH-CRECL-9	1	1	0.125	**8**	8	8	0.25	**32**
TH-CRECL-10	1	1	0.25	**4**	4	4	0.5	**8**
TH-CRECL-11	1	1	0.25	**4**	8	8	0.5	**16**
TH-CRECL-12	0.5	0.5	0.125	**4**	4	4	0.125	**32**
TH-CRECL-13	2	2	1	2	8	8	4	2
TH-CRECL-14	2	2	0.125	**16**	8	8	0.125	**64**
TH-CRECL-15	0.5	0.5	0.125	**4**	4	4	0.25	**16**
TH-CRECL-16	0.5	0.5	0.125	4	4	4	2	2
TH-CRECL-17	0.5	0.5	0.125	4	4	4	2	2
TH-CRECL-18	0.5	0.5	0.125	**4**	4	4	0.25	**16**
TH-CRECL-19	2	2	1	2	8	8	4	2
TH-CRECL-20	0.5	0.5	0.25	2	4	4	2	2
TH-CRECL-21	1	1	0.125	**8**	8	8	0.5	**16**
TH-CRECL-22	2	2	0.25	**8**	8	8	0.5	**16**
TH-CRECL-23	2	2	0.5	**4**	8	8	2	**4**
TH-CRECL-24	0.5	0.5	0.125	**4**	8	8	0.25	**32**
TH-CRECL-25	2	2	1	2	4	4	4	1
TH-CRECL-26	1	1	0.5	2	8	8	4	2
TH-CRECL-27	2	2	1	2	4	4	2	2
TH-CRECL-28	0.25	0.25	0.25	1	4	4	2	2
ATCC13037	0.5	0.5	0.25	2	NA	NA	NA	NA

Notes: Boldface indicate that efflux pump inhibitor assay have positive results based on the MIC decrease to a quarter at least.

^a^TIC, tigecycline MIC(mg/L).

^b^TIG+CCCP, tigecycline + carbonyl cyanide 3-chlorophenylhydrazone, MIC(mg/L); TIG + PAβN, tigecycline + Phe-Arg-β-naphthylamide, MIC(mg/L).

^c^Fold-change, tigecycline MIC decreased fold change under the pressure of PAβN.

^d^Colonies selected from highest tigecycline concentration agar plants in PAPs test.

### Clinical features of TH-CRECL isolates

**Figure 3.**
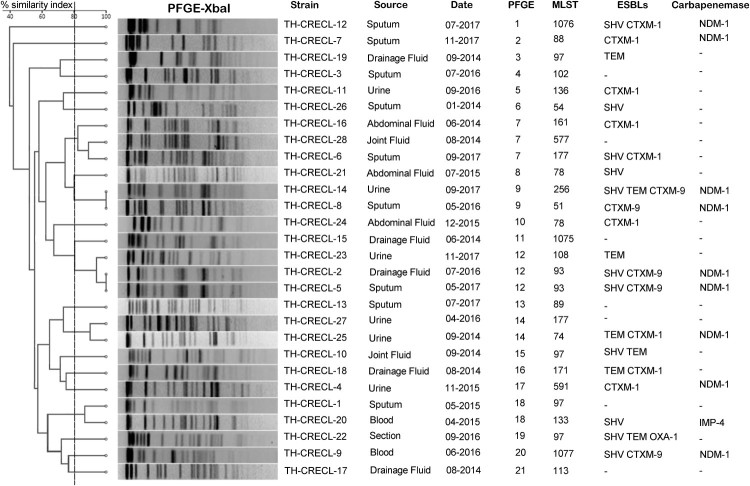
PFGE-based dendrogram of tigecycline-heteroresistant and carbapenem-resistant *E. cloacae* strains. Strain numbers, source of initial isolation, and demographic information are included along each PFGE lane. MLST, multilocus sequence typing; ESBLs, Extended Spectrum Beta-Lactamases; “-”, not detected.

Among 140 non-duplicate *E. cloacae* strains, 28 isolates (28/140; 20.0%) were identified as heteroresistance to tigecycline and distributed sporadically each year (Figure 3). More than one-third of the TH-CRECL isolates were isolated from drainage liquid (10/28; 35.7%), followed by sputum (8/28; 28.6%), urine (7/28; 25.0%), blood (2/28; 7.1%) and wound section (1/28; 3.6%). In addition, tigecycline heteroresistant strains exhibited variable susceptibility to other antimicrobials: highly resistant to ceftazidime (28/28, 100%), ceftriaxone (28/28, 78.6%), cefepime (17/28, 60.7%), gentamicin (13/28, 46.4%), tobramycin (10/28, 35.7%), ciprofloxacin (16/28, 57.1%) levofloxacin (13/28, 46.4%), whereas low level of resistance to amikacin (3/28, 10.7%) and colistin (4/28, 14.3%) as shown in Table S2. Twenty-four (85.7%) TH-CRELC strains exhibited multidrug-resistant traits. Moreover, the prevalence of ESBLs gene and carbapenemase gene in TH-CRECL strain were 75.0% (21/28) and 35.7% (10/28), respectively (Figure 3). It is worth noting that all harboured carbapenemase genes isolates were the positive of β-lactamases.

### Molecular epidemiology of TH-CRECL strains

The detailed characteristics of the molecular epidemiology of the TH-CRECL strains were displayed in Figure 3. The molecular typing of all the TH-CRECL isolates was grouped into 21 different PFGE patterns. MLST results demonstrated that ST97 was the most prevalent genotype in all TH-CRECL isolates (4/28, 14.3%), followed by ST177 (3/28, 10.7%) and ST93 (2/27, 7.14%). Of interest, novel ST1075, ST1076 and ST1077 were found and submitted to the MLST database. Combined with results of PFGE and MLST, no strong epidemiological relationship among these TH-CRECL isolates.

### Molecular mechanisms of TH-CRECL strains

**Figure 4.**
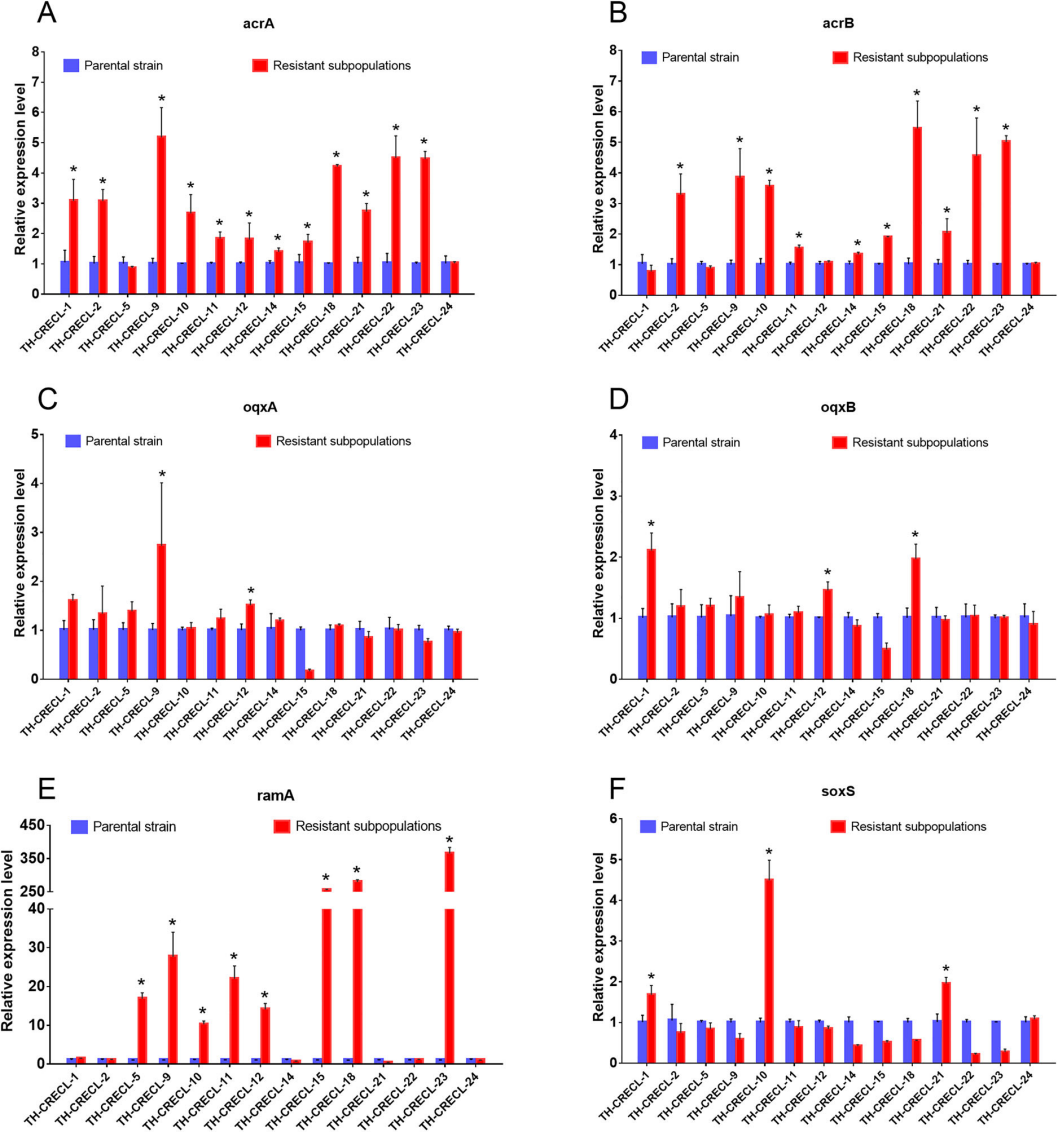
Relative expression level of efflux pump genes and their regulator genes in tigecycline-heteroresistant and carbapenem-resistant *E. cloacae.* Relative expression of each target gene in subpopulations of resistance was compared with the corresponding gene expression of the respective native populations that were used as controls (expression = 1). “*”, Statistically significant (*P* < 0.05).

After the addition of CCCP (Carbonyl cyanide 3-chlorophenylhydrazone), the tigecycline MICs of the heteroresistant isolates and their resistant subpopulations were not significantly reduced, and the reference isolate was also unaffected either (Table 1). However, compared to absence of any EPI (Efflux pump inhibitors), we had detected the tigecycline MICs reduced at least four-fold by the addition of PAβN (Phe-Arg-β-naphthylamide), in 60.7% (17/28) of the TH-CRECL parental strains and 50% (14/28) of their resistant colonies, respectively (Table 1). Moreover, all the resistant subpopulations of positive EPI results exhibited similar inhibitory effects of the native strains after exposure to PAβN. The relative expression of the RND efflux pump genes and their regulators were presented in Figure 4. Significant upregulations of *acrA* (1.40–5.18 folds) or *acrB* (1.33–5.45 folds) were detected in 78.6% (11/14) TH-CRECL isolates. Compared with the original strains, significantly overexpressed the mRNA levels of *oqxA* (1.51–3.42 folds) or *oqxB* (1.46–2.11 folds) were observed in 28.6% (4/14) TH-CRECL resistant subpopulations. Regarding efflux pump regulator genes, we found that 57.1% (8/14) of TH-CRECL isolates were the presence of upregulated *ramA* gene (10.24–356.7 folds), while three TH-CRECL isolates were observed overexpression of *soxS gene* in their resistant subpopulations (1.95–4.49 folds). Notably, AcrAB efflux pump might be more common related to tigecycline heteroresistance than the oqxAB efflux pump (78.6% vs. 28.6%; *P* = 0.021). Only one TH-CRECL isolate was not overexpressed any efflux pump genes and their regulator genes. Overall, these data suggested that upregulations of RND efflux system were significantly associated with tigecycline heteroresistance in CRECL isolates.

### Potential risk factors and clinical outcomes of TH-CRECL infections

The potential factors and outcomes of patients with TH-CRECL infections were shown in Table 2. The univariate analysis indicated that patients with transferring from other hospital, admission to ICU, solid malignancies, respiratory diseases, respiratory infections, tracheal intubation, and previous application of fluoroquinolone were more vulnerable to have the TH-CRECL infection (*P *< 0.05). In multivariate logistic regression analysis, previous fluoroquinolone therapy within 3 months (OR [Odd ratio] = 6.516, 95% CI [Confidence Interval] = 2.159–19.665; *P* < 0.001) was the only factor for the acquisition of TH-CRECL strains (Table 3). We found a significant difference in overall length of hospital stay (28.3 days vs. 23.4 days; *P* = 0.009) and status deterioration (OR = 3.046, 95% CI = 1.117–8.307; *P* = 0.037) between cases and controls. Additionally, three cases (10.7%) with TH-CRECL infection died in this study, but no significant difference in hospital mortality was observed between the two groups.

**Table 2.** Univariate analysis of clinical features of patients infected with tigecycline-heteroresistant and carbapenem-resistant *E. cloacae* isolates.

Characteristics and variables	TH-CRECL	NTH-CRECL	Univariate analysis	
	*n* = 28	*n* = 112	*P*-value^a^	OR (95%CI)
*Demographics*				
Elderly (≥60 years)	16 (57.1)	62 (55.4)	1.000	1.075 (0.466–2.481)
Male gender	19 (67.9)	71 (63.4)	0.826	1.219 (0.505–2.943)
Transfer from other hospital	13 (46.4)	24 (21.4)	**0.015**	3.178 (1.378–8.346)
30-day readmission	1 (3.6)	11 (9.8)	0.459	0.340 (0.042–2.751)
Admission to ICU	18 (64.3)	46 (41.1)	**0.034**	2.583 (1.093–6.102)
*Underlying diseases and conditions*				
Hypertension	11 (39.3)	42 (37.5)	1.000	1.078 (0.461–2.522)
Diabetes mellitus	6 (21.4)	21 (18.8)	0.790	1.182 (0.426–3.277)
Cardiovascular disease	5 (17.9)	11 (9.8)	0.315	1.996 (0.632–6.304)
Neurological disease	2 (7.1)	14 (12.5)	0.739	0.538 (0.115–2.520)
Solid malignancy	8 (28.6)	13 (11.6)	**0.037**	3.046 (1.117–8.307)
Chronic kidney disease	9 (32.1)	26 (23.2)	0.337	1.567 (0.633–3.878)
Hepatobiliary disease	2 (7.1)	24 (21.4)	0.105	0.282 (0.062–1.273)
Gastrointestinal disease	3 (10.7)	24 (21.4)	0.286	0.440 (0.122–1.582)
Respiratory diseases	15 (53.9)	29 (25.9)	**0.007**	3.302 (1.405–7.762)
Endocrine, metabolic disease	1 (3.6)	8 (7.1)	0.687	0.481 (0.058–4.017)
Vascular, hematological disease	1 (3.6)	6 (5.4)	1.000	0.654 (0.076–5.667)
Immunosuppressive state^b^	11 (39.3)	47 (42)	0.834	0.895 (0.384–2.086)
Skin infection	2 (7.1)	16 (14.3)	0.527	0.462 (0.100–2.137)
Urinary tract infection	6 (21.4)	12 (10.7)	0.202	2.273 (0.262–3.815)
Respiratory infection	14 (50.0)	27 (24.1)	**0.010**	3.148 (1.335–7.425)
APACHE II score, median (IQR)^c^	21.7 (15.5–24.0)	19.0 (14.0–22.0)	0.107	NA
*Invasive procedures within prior 4 weeks*				
Surgery in the past 6 months	18 (64.3)	58 (51.8)	0.291	1.676 (0.769–6.714)
Receipt of total parenteral nutrition	8 (28.6)	26 (23.2)	0.623	1.323 (0.522–3.353)
Mechanical ventilation	10 (35.7)	36 (32.1)	0.822	1.173 (0.492–2.796)
Bladder irrigation	3 (10.7)	11 (9.8)	1.000	1.102 (0.286–4.248)
Drainage tube	13 (46.3)	63 (56.3)	0.400	0.674 (0.294–1.548)
Urinary catheter	19 (67.9)	63 (56.3)	0.292	1.642 (0.683–3.945)
Tracheal cannula	16 (57.1)	28 (25.0)	**0.002**	4.000 (1.689–9.472)
Nasal catheter	6 (21.4)	14 (12.5)	0.235	1.909 (0.660–5.523)
Central venous catheter	12 (42.9)	44 (39.3)	0.830	1.159 (0.501–2.682)
*Antimicrobial exposure within 3 months*				
cephalosporins	20 (71.4)	84 (75)	0.809	0.833 (0.331–2.101)
Carbapenem	10 (37.5)	37 (33)	0.825	1.126 (0.473–2.681)
Aminoglycosides	5 (17.9)	12 (10.7)	0.334	1.812 (0.581–5.650)
Fluoroquinolone	12 (42.9)	12 (10.7)	**<0.001**	6.250 (2.397–16.299)
Tetracycline	5 (17.9)	8 (7.1)	0.136	2.826 (0.847–9.432)
Macrolides	2 (7.1)	9 (8.1)	1.000	0.880 (0.179–4.323)
Metronidazole	5 (17.9)	36 (32.1)	0.168	0.459 (0.161–1.305)
Glycopeptide	5 (17.9)	12 (10.7)	0.334	1.812 (0.581–5.650)
Antifungal agents	3 (10.7)	14 (12.5)	1.000	0.840 (0.224–3.151)
Combined use of antibiotics	15 (53.6)	59 (52.7)	1.000	1.037 (0.452–2.377)
*Clinical outcomes*				
In-hospital mortality	3 (10.7)	9 (8.0)	0.706	1.373 (0.346–5.447)
Functional status deterioration	8 (28.6)	13 (11.6)	**0.037**	3.046 (1.117–8.307)
Postculture length of hospital stay, median (IQR), days	16.6 (8.3–22.8)	15.0 (9.0–20.8)	0.474	NA
Total length of hospital stay, median (IQR), days	28.3 (20.8–35.8)	23.4 (16.3–30.0)	**0.009**	NA

Notes: Data are expressed as no. (%) unless specified otherwise. OR, odds ratio; CI, confidence interval; ICU, intensive care unit; IQR, interquartile range; NA, not available.

^a^Boldface indicate values that are significant (*P* < 0.05).

^b^Immunosuppression was defined as meeting one of the following criteria: severe anemia, hypoalbuminaemia, neutropenia, or receiving chemotherapy.

^c^APACHE: Acute Physiology and Chronic Health Evaluation. The APACHE II score was calculated from worst values of physiological variables, which were obtained in the first 24 h of ICU admission.

**Table 3.** Multivariable model of risk factors associated with tigecycline-heteroresistance among carbapenem-resistant *E. cloacae* isolates.

Characteristics and variables	TH-CRECL	NTH-CRECL	Multivariable analysis	
	*n* = 28	*n* = 112	*P*-value^a^	OR (95%CI)
Transfer from other hospital	13 (46.4)	24 (21.4)	0.109	2.283 (0.831–6.275)
Admission to ICU	18 (64.3)	46 (41.1)	0.714	1.250 (0.378–4.138)
Solid malignancy	8 (28.6)	13 (11.6)	0.113	2.585 (0.799–8.367)
Respiratory diseases	15 (53.9)	29 (25.9)	0.109	2.282 (0.831–6.266)
Respiratory infection	14 (50.0)	27 (24.1)	0.137	2.255 (0.772–6.591)
Tracheal cannula	16 (57.1)	28 (25.0)	0.337	1.814 (0.538–6.121)
Fluoroquinolone	12 (42.9)	12 (10.7)	**<0.001**	6.516 (2.159–19.665)

Notes: Data are no. (%) unless specified otherwise. OR, odds ratio; CI, confidence interval; ICU, intensive care unit; IQR, interquartile range; NA, not available.

^a^Boldface indicate values that are significant (*P* < 0.05).

## Discussion

Heteroresistance described a phenomenon where population-wide within a pathogen exhibited diverse susceptibilities to a certain antibiotic, which was considered as a preliminary stage for entirely evolving from susceptibility to resistance [22]. In the present study, our results showed that 20.0% of clinical carbapenem-resistant *E. cloacae* isolates were identified as tigecycline heteroresistance, which was relatively lower than our previous reports on carbapenem heteroresistance in *E. coli* (34.3%) and *Pseudomonas aeruginosa* (84.9%) [14,16]. The most likely explanation for this difference, at least partially, was that tigecycline was not the usual drugs used in some serious and complicated salvage therapy in our hospital. On the other hand, the additive structure of minocycline molecule provided the tigecycline the capability to overcome the classical resistance mechanisms and reduce susceptibility to the antibiotic resistance development [23]. Additionally, molecular epidemiological analysis has ruled out the potential outbreak of these TH-CRECL strains. Moreover, antibiotic susceptibility data and resistance genes amplification confirmed that the TH-CRECL strains have multidrug-resistant profiles, suggesting that the emergence of tigecycline heteroresistance might further reduce the therapeutic choices in CRE strains.

A recent study on *Salmonella enterica* has shown that tigecycline heteroresistance was related to the upregulated AcrAB and OqxAB efflux systems [24]. Consistent with results from the above-mentioned report, we observed that resistant subpopulations in tigecycline heteroresistance in carbapenem-resistant *E. cloacae* displayed a significant overexpression of AcrAB and/or OqxAB efflux pumps compared to their respective native isolates. The efflux pump regulators *ramA* and/or *soxS* were showed the increased mRNA level in resistant subpopulations of heteroresistant strains, in line with a previous study on the tigecycline resistance mechanism [25]. One possible explanation for this finding might be that the overexpressed AcrAB-TolC complex extruded a variety of substrate specificity, and decreased the susceptibility to most antibiotics [26]. Interestingly, we observed one strain with the reduced MIC levels under pressure of PaβN, but not showed the overexpression of any efflux pumps and regulators, possibly due to other RND efflux systems might be involved in the procession of tigecycline heteroresistance.

In contrast to the *in vitro* evolution of resistance predating use of tigecycline in *Klebsiella pneumonia* [9] and *E. coli* [27], our study found that all the patients with TH-CRECL infections did not receive any tigecycline treatment. Interestingly, our finding demonstrated that previous fluoroquinolone therapy within three months increased the possibility of acquiring the TH-CRECL infection. It is reasonable to posit that fluoroquinolones are transferred by the same efflux systems (AcrAB and OqxAB) as tigecycline, which could facilitate the upregulation of RND efflux pumps and enhance the ability of tigecycline export, thus making the bacteria survive in the higher concentrations of tigecycline. Furthermore, *E. cloacae* isolates with overexpressed AcrAB efflux pump displayed a MDR phenotype [11,28], which may be a possible explanation for TH-CRECL bacteria cross-resistance to multiple other antibiotics.

Surprisingly, our study revealed that patients infected with TH-CRECL strains showed significantly longer stay of hospitalization and poorer clinical outcomes than patients with NTH-CRECL infection. One possible explanation for this finding might be the overexpressed efflux pumps of TH-CRECL strains significantly increased the antimicrobial resistance and showed the bacterial multidrug resistance phenotype, thus leading the patients to a longer duration of hospitalization and higher morbidity [29,30]. Another possible explanation could be that RND-type efflux pumps were involved in the virulence factors production and biofilm formation, resulting in some serious diseases or invasive infections [31,32]*.* However, in-hospital mortality was no statistical significance, probably due to the limited number of cases with TH-CRECL infection in this study.

Regarding the treatment of heteroresistant infection, one previous study revealed that colistin heteroresistance could be inhibited by using colistin in combination with tigecycline or rifampin [33]. Moreover, combinations of some active antibiotics were also significantly associated with a reduced death in CRE patients [34]. Given that amikacin and colistin display the relative excellent activities against TH-CRECL pathogens in our study, it is reasonable to consider the combination regimens of amikacin (or colistin) with tigecycline to prevent the resistant subpopulations in these isolates.

It should be highlighted the importance of screening for the heteroresistance in clinical isolates. For the treatment of CRE infection, we recommended the routine screening of disk diffusion or E-test to detect the tigecycline heteroresistance in CRE isolates. Moreover, clinicians and microbiologists should be aware of the underlying threaten of this new emerging phenomenon. It is a critical need to timely detect and improve identification of the heteroresistant strains and further optimize combination therapies to eradicate them.

This study has some limitations. First, our analysis has the limited sample size for this new emerging phenomenon. However, the clinical impact of tigecycline heteroresistance in CRE is of great importance to antibiotic treatment, thus needing a special concern. Second, due to the findings of this study based on the statistical inference and hypothesis, subsequent research should exhaustively explore the functions of RND efflux system in tigecycline heteroresistance.

In conclusion, our study for the first time described the clinical and molecular characteristics of tigecycline heteroresistant in carbapenem-resistant *E. cloacae*. Mechanistic analysis revealed that this phenomenon could be associated with the overexpression of AcrAB and OqxAB efflux pumps. In addition, previous fluoroquinolone therapy was identified as the only predictor for the acquisition of TH-CRECL strains. For clinical outcomes, patients infected with TH-CRECL strains might lead to a longer hospital stay and a worse deterioration. Our future study will be focused on the screening some new antibiotics for monotherapy or combinations to intervene the heteroresistance development.

## Supplementary Material

Supplemental Material
