# Supplementary material for: Detection and characterization of tigecycline heteroresistance in E. cloacae: clinical and microbiological findings
Source: Emerg Microbes Infect. 2019 Apr 4;8(1):564–74. doi: 10.1080/22221751.2019.1601031 (PMC6455127; doi:10.1080/22221751.2019.1601031)
Supplement: Supplemental Material [file TEMI_A_1601031_SM5241.docx]

**Table S1** Primers used for qRT-PCR

| **Target** | **Primer** | **Sequence (5'-3')** | **Size** | **Source or Ref.** |
| --- | --- | --- | --- | --- |
| ***acrA*** | acrA-F | GCCTCTGGCGGTCGTTCTGAT | 126bp | Ref. 11 |
|  | acrA-R | AGAGGTTCGGATTTGAGCGTCAC |  |  |
| ***acrB*** | acrB-F | GTGAGCGTCGAGAAATCGTCCA | 127bp | Ref. 11 |
|  | acrB-R | TACGGCTGATGGCGTCCTTCAT |  |  |
| ***oqxA*** | oqxA-F | TATCGCGCCACGCTGGAACAGG | 140bp | This study |
|  | oqxA-R | CGGCGCTGCTCCCACTCTTC |  |  |
| ***oqxB*** | oqxB-F | GATCAGGCGCAGGTTCAGGTGCA | 128bp | This study |
|  | oqxB-R | AACAGATGCACCACCAGCGTCAG |  |  |
| ***ramA*** | ramA-F | CGCTCAGGTCATTGATACGA | 144bp | Ref. 12 |
|  | ramA-R | CCCAGACTTTCGCCTTTGTA |  |  |
| ***soxS*** | soxS-F | GTCGCATCAGCAAATTATTCAGAC | 128bp | This study |
|  | soxS-R | TGCGGAACATTCTCTGTAAATACC |  |  |
| ***rpoB*** | rpoB-F | AAGGCGAATCCAGCTTGTTCAGC | 148bp | Ref. 25 |
|  | rpoB-R | TGACGTTGCATGTTCGCACCCATCA |  |  |

**Table S2** Minimum inhibitory concentrations (MICs) of different antibiotics in tigecycline-heteroresistant and carbapenem-resistant *E. cloacae* isolates.

| Isolate | CAZ | CRO | FEP | ETP | IPM | ATM | GEN | TOB | AMK | CIP | LVX | COL |
| --- | --- | --- | --- | --- | --- | --- | --- | --- | --- | --- | --- | --- |
| TH-CRECL-1 | ≥64 | ≥64 | 8 | 2 | ≤1 | ≥64 | ≥16 | ≥16 | 32 | ≥4 | ≥8 | 4 |
| TH-CRECL-2 | ≥64 | ≥64 | ≥64 | ≥8 | 8 | ≥64 | ≥16 | ≥16 | ≤2 | ≥4 | ≥8 | 1 |
| TH-CRECL-3 | ≥64 | ≥64 | 4 | 4 | ≤1 | ≥64 | ≤1 | 4 | ≤2 | ≥4 | 4 | 1 |
| TH-CRECL-4 | ≥64 | ≥64 | 4 | >16 | >8 | ≥64 | ≥16 | 8 | ≤2 | 1 | 1 | 1 |
| TH-CRECL-5 | ≥64 | ≥64 | ≥64 | ≥8 | ≥16 | ≥64 | ≥16 | ≥16 | ≤2 | ≥4 | ≥8 | 1 |
| TH-CRECL-6 | ≥64 | ≥64 | 2 | 2 | ≤1 | ≥64 | ≤1 | ≤1 | ≤2 | ≤0.25 | ≤0.25 | 2 |
| TH-CRECL-7 | ≥64 | ≥64 | 32 | 8 | 4 | ≥64 | ≤1 | ≤1 | ≤2 | ≤0.25 | ≤0.25 | 2 |
| TH-CRECL-8 | ≥64 | ≥64 | ≥64 | ≥8 | ≥16 | ≤1 | ≤1 | ≤1 | ≤2 | ≥4 | ≥8 | 2 |
| TH-CRECL-9 | ≥64 | ≥64 | ≥64 | ≥8 | 8 | ≥64 | ≥16 | ≥16 | ≤2 | ≥4 | ≥8 | 1 |
| TH-CRECL-10 | ≥64 | ≥64 | ≥64 | 4 | 2 | ≥64 | ≥16 | ≥16 | 32 | ≥4 | ≥8 | 1 |
| TH-CRECL-11 | ≥64 | ≥64 | ≥64 | 2 | ≤1 | ≥64 | ≥16 | ≥16 | 8 | ≥4 | ≥8 | 8 |
| TH-CRECL-12 | ≥64 | ≥64 | ≤1 | ≥8 | 8 | ≥64 | ≤1 | ≤1 | ≤2 | ≤0.25 | ≤0.25 | 2 |
| TH-CRECL-13 | ≥64 | ≥64 | 32 | 2 | ≤1 | ≥64 | ≥16 | ≥16 | ≤2 | ≥4 | 4 | 1 |
| TH-CRECL-14 | ≥64 | ≥64 | ≥64 | ≥8 | ≥16 | ≥64 | ≥16 | 8 | ≤2 | ≥4 | ≥8 | 1 |
| TH-CRECL-15 | ≥64 | ≥64 | ≤1 | 2 | ≤1 | ≤1 | ≤1 | ≤1 | ≤2 | ≤0.25 | ≤0.25 | 8 |
| TH-CRECL-16 | ≥64 | ≥64 | ≤1 | 2 | 2 | 16 | ≤1 | ≤1 | ≤2 | ≤0.25 | ≤0.25 | 1 |
| TH-CRECL-17 | ≥64 | 8 | ≤1 | 2 | ≤1 | ≥64 | ≥16 | ≥16 | 16 | ≥4 | ≥8 | 2 |
| TH-CRECL-18 | ≥64 | ≥64 | ≥64 | 4 | ≤1 | ≥64 | ≥16 | ≥16 | ≥64 | ≥4 | ≥8 | 1 |
| TH-CRECL-19 | ≥64 | ≥64 | ≥64 | 2 | ≤1 | ≤1 | ≤1 | ≤1 | ≤2 | 2 | 2 | 4 |
| TH-CRECL-20 | ≥64 | ≥64 | 32 | ≥8 | ≥16 | ≤1 | ≤1 | ≤1 | ≤2 | 1 | 1 | 1 |
| TH-CRECL-21 | ≥64 | ≥64 | ≥64 | 2 | ≤1 | ≤1 | ≤1 | ≤1 | ≤2 | ≤0.25 | ≤0.25 | 2 |
| TH-CRECL-22 | ≥64 | ≥64 | ≥64 | 4 | ≤1 | ≥64 | 8 | 8 | ≤2 | ≥4 | ≥8 | 1 |
| TH-CRECL-23 | ≥64 | ≥64 | ≤1 | 2 | 2 | ≤1 | ≥16 | 8 | ≤2 | ≥4 | 1 | 1 |
| TH-CRECL-24 | ≥64 | ≥64 | ≥64 | 4 | ≤1 | ≥64 | 8 | 8 | ≤2 | ≥4 | ≥8 | 1 |
| TH-CRECL-25 | ≥64 | ≥64 | ≥64 | ≥8 | ≥16 | ≥64 | ≥16 | 2 | ≤2 | ≥4 | ≥8 | 1 |
| TH-CRECL-26 | ≥64 | ≥64 | 2 | 4 | ≤1 | ≥64 | ≤1 | ≤1 | ≤2 | ≤0.25 | ≤0.25 | 2 |
| TH-CRECL-27 | ≥64 | ≥64 | ≥64 | 4 | 2 | ≥64 | 8 | ≥16 | 16 | ≤0.25 | 1 | 2 |
| TH-CRECL-28 | ≥64 | ≥64 | ≤1 | 2 | ≤1 | ≤1 | ≤1 | ≤1 | ≤2 | ≤0.25 | ≤0.25 | 2 |

CAZ, ceftazidime; CRO, ceftriaxone; FEP, cefepime; ETP, ertapenem; IMP, imipenem; ATM, aztreonam; GEN, gentamicin; TOB, tobramycin; AMK, amikacin; CIP, ciprofloxacin; LEV, levofloxacin; COL, colistin;
